# Alcohol in Neurasthenia

**Published:** 1894-11

**Authors:** Græme M. Hammond

**Affiliations:** New York


ALCOHOL IN NEURASTHENIA. By Græme M. Hammond, M. D., New York.—The diet to be observed in neurasthenia is a question which deserves a great deal of careful consideration. In many cases the digestive organs fail to perform their functions properly, either because the digestive juices are not secreted in their proper proportion, or else chemical changes in their composition diminish or interfere with their activity. This results generally in quantative indigestion, that is the inability to digest more than a very limited quantity of food, but sometimes certain classes of foods seem to be discriminated against much more than others.

It is not my purpose in this article to consider the subject of digestion in neurasthenia in all its aspects, but to confine myself solely to the influence of alcohol on the digestion of the neurasthenic and on the neurasthenia itself.

The free use of alcohol is always more or less injurious to the normal individual, but it is particularly so in cases of neurasthenia. Patients of this description usually find out for themselves that the free indulgence in wines aggravates their headaches, increases their insomnia, induces more indigestion than they usually have and augments their general symptom of discomfort. On the other hand, it has been my experience that small quantities of alcohol, given with the heaviest meal, frequently assists a feeble digestion. More than this, it seems to dissipate, for a time at least, the depression and confusion which are so often prominent symptoms. It is true that alcohol retards the action of pepsin in experiments performed outside of the body, but within the stomach diluted alcohol, in small quantities, seems to stimulate the gastric tubules and thus increases the secretion of gastric juice. It is the function of the gastric juice to conxert proteids or nitrogenous food into peptones. A diminished quantity of gastric juice, therefore, delays or arrests the digestion of meats, albumin and gelatinous foods, all of which are nitrogenous and, as a class, are very necessary in supplying muscular strength and vitality. The gastric irritation consequent upon indigestion has in itself a depressing effect upon the nervous system. It has long been my custom, therefore, to advocate the ingestion of a small quantity of alcohol in the form of a glass of claret with the patient's heaviest meal. Of recent years I have used one or more of the various preparations of wine of coca, as it seemed to me the tonic and stimulating effects of the coca on the nervous system, together with the gastric stimulation from the small quantity of alcohol, had generally a more beneficial effect than claret alone. More recently I have used maltine with coca wine. Here the maltine, which contains diastase, materially aids in the digestion of the starchy foods, while the small quantity of alcohol it contains stimulates the secretion of gastric juice and thus assists in the digestion of the nitrogenous substance. On the other hand, the coca acts as a mild tonic and stimulant to the nervous system, diminishing the irritability and despondency and promoting the gradual restoration of nervous strength. Maltine with coca wine is a preparation agreeable to the palate, is a food in itself, assists in the digestion of starchy and nitrogenous foods, and is also a useful tonic to the nervous system. In this form moderate quantities of alcohol can be administered to the best advantage.—*Journal of Nervous and Mental Disease.*

